# Evidence for the Association between the Intronic Haplotypes of Ionotropic Glutamate Receptors and First-Episode Schizophrenia

**DOI:** 10.3390/jpm11121250

**Published:** 2021-11-25

**Authors:** Katerina Hirschfeldova, Jiri Cerny, Paulina Bozikova, Viktor Kuchtiak, Tobias Rausch, Vladimir Benes, Filip Spaniel, David Gregus, Jiri Horacek, Ladislav Vyklicky, Ales Balik

**Affiliations:** 1 Institute of Biology and Medical Genetics, First Faculty of Medicine, Charles University, 12800 Prague, Czech Republic; khirs@lf1.cuni.cz; 2 Institute of Physiology, Czech Academy of Sciences, 14220 Prague, Czech Republic; jiri.cerny@ibt.cas.cz (J.C.); viktor.kuchtiak@fgu.cas.cz (V.K.); ladislav.vyklicky@fgu.cas.cz (L.V.); 3 Institute of Biotechnology, Czech Academy of Sciences, BIOCEV, 25250 Vestec, Czech Republic; paulina.bozikova@ibt.cas.cz; 4 Faculty of Science, Charles University, 12800 Prague, Czech Republic; 5 Genomics Core Facility, EMBL, 69117 Heidelberg, Germany; rausch@embl.de (T.R.); benes@embl.de (V.B.); 6 The National Institute of Mental Health, 25067 Klecany, Czech Republic; filip.spaniel@nudz.cz (F.S.); david.gregus@nudz.cz (D.G.); jiri.horacek@nudz.cz (J.H.); 7 Institute of Physiology, Czech Academy of Sciences, BIOCEV, 25250 Vestec, Czech Republic

**Keywords:** schizophrenia, ionotropic glutamate receptor, intron, genetic variations, SNP, haplotypes, scoring model, BrainAGE

## Abstract

The heritable component of schizophrenia (SCH) as a polygenic trait is represented by numerous variants from a heterogeneous group of genes each contributing a relatively small effect. Various SNPs have already been found and analyzed in genes encoding the NMDAR subunits. However, less is known about genetic variations of genes encoding the AMPA and kainate receptor subunits. We analyzed sixteen iGluR genes in full length to determine the sequence variability of iGluR genes. Our aim was to describe the rate of genetic variability, its distribution, and the co-occurrence of variants and to identify new candidate risk variants or haplotypes. The cumulative effect of genetic risk was then estimated using a simple scoring model. *GRIN2A-B*, *GRIN3A-B*, and *GRIK4* genes showed significantly increased genetic variation in SCH patients. The fixation index statistic revealed eight intronic haplotypes and an additional four intronic SNPs within the sequences of iGluR genes associated with SCH (*p* < 0.05). The haplotypes were used in the proposed simple scoring model and moreover as a test for genetic predisposition to schizophrenia. The positive likelihood ratio for the scoring model test reached 7.11. We also observed 41 protein-altering variants (38 missense variants, four frameshifts, and one nonsense variant) that were not significantly associated with SCH. Our data suggest that some intronic regulatory regions of iGluR genes and their common variability are among the components from which the genetic predisposition to SCH is composed.

## 1. Introduction

Schizophrenia is a severe neuropsychiatric disorder estimated to affect up to 0.5% of the population worldwide [1,2]. The strong genetic component of SCH has been demonstrated in studies of twins or affected families [3]. The disorder is characterized by positive symptoms, negative symptoms, and cognitive deficits [4,5]. Several lines of evidence, such as altered mRNA and protein expression or de novo mutations in protein-coding regions of iGluR genes [6,7,8], have supported the hypothesis of dysfunction of glutamate-dependent excitatory signaling in the development of SCH.

Excitatory signaling in the central nervous system is mediated by ionotropic glutamate receptors (iGluR), the ion channels which are divided pharmacologically into three subclasses: AMPA, kainate (KA), and NMDA receptors [9,10,11]. The iGluR family consists of sixteen genes encoding four AMPAR (GRIA1-4), five KAR (GRIK1-5), and seven NMDAR subunits (GRIN1, 2A-D, 3A-B) [12]. AMPA and KA receptors form homo- or heterotetramers [13,14]. In contrast, NMDAR is strictly a heterotetramer composed of two obligatory GluN1 subunits and two GluN2 or GluN3 subunits in different combinations [15,16]. The subunit composition determines the specific functional properties of each iGluR including its intracellular localization and trafficking to the synapse [9]. The AMPA and NMDA receptors located on the postsynaptic membrane are involved in excitatory signal transduction upon glutamate release into the synaptic cleft. The physiological function of AMPA and NMDA receptors is fundamental for learning, cognition, memory formation processes, and behavior [17,18]. The behavioral and cognitive defects are manifested in various neurodevelopmental disorders [19].

Previous pharmacological studies have shown that NMDAR antagonists can induce psychotic symptoms typical of SCH in healthy individuals and significantly exacerbate these symptoms in patients [20,21]. Therefore, NMDAR hypofunction is thought to play a role in the etiology of SCH. Furthermore, in genetically modified mice, altered expression of the GluN1 subunit to 5% of its physiological level resulted in hypofunctional glutamatergic signaling. GluN1-deficient mice exhibited behavioral deficits that mimicked pharmacologically induced SCH symptoms [22]. In addition, several genetic variations have been identified in the coding and noncoding regions of NMDAR genes. However, their association with SCH was low or limited to a specific group of patients. Most SNPs in NMDAR exons are synonymous and rare mutations [23,24,25,26,27,28,29,30,31]. Other variations have also been identified in the untranslated and promoter regions of NMDAR genes where they affect gene expression as determined by the in vitro luciferase assay [32,33]. In the last decade, genome-wide association studies underlined the polygenic nature of SCH with multiple risk variants involved [34,35,36]. Consistent with previous single gene studies, a number of identified genetic variants has been found in genes that form the signaling complex of NMDAR, including the postsynaptic complex PSD-95 or Arc gene [37,38,39,40]. A global study identified 108 gene loci associated with SCH, including loci harboring genes for ionotropic GluA1 and GluN2A. This work confirmed many previous studies indicating genetic alterations in genes involved in excitatory glutamatergic signaling [34]. 

The exonic mutations in iGluR genes previously found in SCH were rare or de novo and could contribute only to specific cases. However, an increased number of small-risk alleles in regulatory and intronic parts of iGluR genes, if accumulated, could be responsible for hypofunction of the glutamatergic system in patients. 

To evaluate this possibility in this case–control study, we used the gene-targeted sequencing strategy and resequenced all sixteen iGluR genes. In addition to exonic mutations, we focused on searching for variations in noncoding parts of these genes to predict their possible effects on gene expression and the extent of their association with SCH and its brain morphological phenotype (gray matter volume). To test the cumulative effect of small-risk alleles in our cohort of SCH cases, we proposed a simple scoring model. 

## 2. Materials and Methods

### 2.1. SCH Patients/Control Subjects 

In this case–control study, sixty-three patients with first-episode schizophrenia-spectrum disorders (FES) (aged 24.6 ± 6.9 years) were diagnosed on the basis of the structured MINI International Neuropsychiatric Interview [41] and according to the ICD-10 criteria by two independent psychiatrists. FES subjects were diagnosed with schizophrenia and detected through their first hospitalization at the Bohnice psychiatric hospital with the catchment area of 1 million inhabitants living in Prague and the northern part of Central Bohemia. The diagnostic procedures were performed at the Prague psychiatric center, Prague, CR, or at the National Institute of Mental Health (NIMH), Klecany, CR, within the data collection period of 2014–2017. Patients were diagnosed with FES if they met the following criteria: (a) first hospitalization for schizophrenia-spectrum disorders (schizophrenia or acute and transient psychotic disorders) and (b) clinical interview identified the first psychotic and/or prodromal symptoms of psychosis not earlier than 24 months before (mean, 3.61 ± 5.05 months). The Positive and Negative Symptom Scale, PANSS [42], was rated by experienced psychiatrists (interclass correlation coefficient, 0.73; 95% confidence interval, 0.62–0.83). The majority of the patients were treated with atypical antipsychotics (olanzapine, 29; risperidone, 15; quetiapine, 6; amisulpride, 4; clozapine, 4; aripiprazole, 7; ziprasidone, 2; zotepine, 1; haloperidol, 1; levomepromazine, 1), 43 were on monotherapy, 4 were unmediated, and the rest were on a combination of antipsychotics. Thirty-two healthy control subjects had a similar sociodemographic background to the FES subjects, whom they were selected to match for age and sex. In the final sample, the controls had a slightly higher age and number of years of education than the FES subjects (Table 1). The higher number of SCH patients compared to the controls reflects the fact that we expected a more pronounced influence of risk loci on gray matter reduction (BrainAGE) in the SCH group, and the fixation index (Fst) allows us to estimate the portion of genetic variability in the unequally distributed samples.

The controls were screened using MINI and were excluded if they had a (a) lifetime history of any psychiatric disorders or (b) a family history of psychotic disorders up to the second degree (confirmed by diagnostic interviews and electronic medical records). Additional exclusion criteria for both groups were a history of seizures or significant head trauma, mental retardation, a history of substance dependence, and any MRI contraindications. 

### 2.2. Genomic DNA Analysis 

Genomic DNA was extracted from whole blood samples using a QIAamp DNA Blood Kit according to the manufacturer’s protocol. A NimbleGen Custom Sequence Capture Kit was used for ninety-six samples to capture a set of target regions for deep sequencing. The custom design included ~15 Mb of genomic sequence, approximately 0.5% of the human genome, which also targets the complex of all glutamate receptor genes. Briefly, genomic input DNA was sheared to a mean size of 178 bp. The captured fragments from each sample were sequenced as 75–80 bp paired-end reads using an Illumina HiSeq 2500 sequencing instrument at EMBL GeneCore. We used bwa mem [43] for sequence alignment against the genomic reference hg38. The average on-target rate (±100 bp of the target region) was estimated to be 85% using Alfred [44]. The percentage of duplicated reads varied in the range of 10–45% (mean: 24%). After deduplication, almost 90% of the captured targets had a mean coverage ≥ 30x for each sample. Based on the alignments, the sequencing error rate was estimated to be <1% for all the samples. Variant calling was performed using FreeBayes [45], the vcf file was filtered for read depth and mapping quality and finally annotated using VEP [46]. The complete alignment and variant calling workflow is available in the GitHub nRex repository (https://github.com/tobiasrausch/nRex (last accessed on 17 November 2021)). Further filtering based on our case–control design was performed using custom scripts.

### 2.3. Sequencing Data Analysis

Sixteen genes encoding ionotropic glutamate receptors span over 4.2 Mb and contain extended promoter regions (characterized as 1500 bp upstream of the canonical transcription start site, TSS), 5′UTRs, all coding parts of exons, intron regions, and a 3′UTRs region of variable length. The genomic coordinates of these regions were assigned according to the GENCODE annotation (Ensembl/Havana with consensus coding sequences selected according to CCDS) and saved as a BED file. Genomic variants within each region were then extracted and analyzed using the bcftools program from the merged regenotyped BCF-formatted file generated by nRex. All the identified variants were annotated based on the local copy of the Kaviar (Known VARiants) database and by Ensembl VEP. The distribution of variants was referenced to the canonical transcripts. For *GRIA4* and *GRIK1* where the TSL method for highlighting a well-supported model of transcript structure does not support a single transcript model, the canonical transcript was defined as the one with the longest coding length. Furthermore, annotation of variants identified within promoter regions was performed using the bedtools intersect with the wgEncode DNaseI Hypersensitivity Clusters and Transcription Factor ChIP-seq data. All necessary transformations of the data and processing of the intermediate results were performed using in-house scripts.

### 2.4. Homology Modeling

Models of the extracellular and transmembrane domains of human GluA2 (P42262), GluK2 (Q13002), and GluN1/GluN2B (Q05586/Q13224) were constructed with the MODELLER 9.14 suite of programs using the available crystal or cryoEM structures. We used structure 5ide as the GluA2 template, structure 5kuf for modeling GluK2, and structures 4pe5, 4tll, and 4tlm for the GluN1/GluN2B model.

### 2.5. Statistical Analysis

To identify candidate loci or chromosomal regions associated with SCH, we used VCFtools to estimate Fst for each autosomal diploid variant (calculated according to the Weir and Cockerham method). Only variants from the last percentile of the Fst distribution corresponding to Fst values above 5% inclusive were analyzed further. The Hardy–Weinberg equilibrium and statistically significant difference in the distribution of selected alleles and haplotypes between cases and controls (Fisher’s exact test) and all other statistical analyses were calculated using Statistica 12. Considering small sample sizes, statistical significance was set at *p*-value < 0.05.

Statistical significance of the case–control data for Manhattan plots was measured using Pearson’s chi-squared test as implemented in the R function chisq.test from the package stats. For a given contingency table (contg_table), we obtained the standardized Pearson residuals, which are residuals adjusted to have asymptotic standard normal distribution, from the R function chisq.test(contg_table)$stdres. The corresponding probability values were computed as chisq.test(contg_table)$*p*.value. 

Pearson’s Chi-square test and the *t*-test were used for sociodemographic data.

### 2.6. Segmentation of the Brain and Estimation of the BrainAGE Index 

Data were acquired on a 3T Siemens Trio MRI scanner (Siemens, Erlangen, Germany) equipped with a standard head coil. For *post hoc* analyses of the influence of gray matter volume, the MRI structural T1-weighted (T1W) 3D-MPRAGE sequence was used: repetition time (TR), 2300 ms; echo time (TE), 4.63 ms; bandwidth, 130 Hz/pixel; field of view (FOV), 256 × 256 mm^2^; matrix, 256 × 256; 160–224 contiguous sagittal slices; voxel size, 1 × 1 × 1 mm^3^; GRAPPA, acceleration factor 2. 

T1W data were segmented using SPM12 (http://www.fil.ion.ucl.ac.uk/spm/soft-ware/spm (accessed on 13 January 2020)) implemented in MATLAB 9.1 (Math Works, Natick, MA, USA). After manual reorientation, T1W images were segmented using the standard unified segmentation model in SPM12. This generated the tissue probability maps reflecting the prior probability of a given voxel belonging to the gray and white matter (GM, WM) and cerebrospinal fluid (CSF). The Diffeomorphic Anatomical Registration Through Exponentiated Lie Algebra (DARTEL) algorithm [41] was used to create a study-specific template to generate GM, WM, and CSF images of each individual spatially normalized in the MNI space. Therefore, the volumes of GM, WM, and CSF were calculated using the MATLAB script get_totals (http://www.cs.ucl.ac.uk/staff/g.ridgway/vbm/get_totals.m (accessed on 14 November 2007)). 

## 3. Results

### 3.1. Genetic Variations in iGluR Genes 

In the canonical transcripts of all sixteen iGluR genes in SCH cases, 16,287 variants were detected, of which 98% were located in the intronic regions. Eighty-one variants were located within the adjacent promoter sequences (defined as 1500 bp upstream of a canonical TSS). A markedly increased number of variants (23%) were found in the SCH subjects in contrast to the controls (Table 2). In both groups, 91% of the variants were SNPs and 9% were insertion–deletion mutations (InDels), with the most common InDel being a single nucleotide deletion/duplication (61%). The vast majority of the variants (97%) found exclusively in SCH were located in intronic sequences, but there was also a substantial number of variants in 3′UTRs and promoters (Supplementary Materials: Supplementary Figure S1). However, most of these variants were rare (minor allele frequency (MAF) ≤ 0.016 in SCH) and randomly distributed. An exception was the GRIN2B 3′UTR variant rs1805503 which was present in six patients but not in the controls. It is a low-conserved nucleotide within a well-conserved segment of the 3′UTR region. The increased number of variants in SCH is due in part to the presence of extensive intronic haplotypes, such as the one we observed in GRIN3A, which spans a distance of 106,796 bp across introns 1 to 5 and consists of 63 SNPs and four InDels. Variants without an assigned reference number were assumed to be novel variants (2.4% in SCH). In 276 detected variants, the alternative allele was present on all chromosomes in both the SCH subjects and the controls and thus represents the ancestral allele in the Czech population.

The highest average variability across all the analyzed regions was found in GRIN2B (7.0 variants per 1000 bp), followed by GRIN2A (5.8), GRIN3A (5.6), GRIN3B (4.9), and GRIK4 (4.2). The lowest variability was found for GRIN1 (1.0) and GRIK5 (1.4). 

One or more upstream ATGs (uATG) are annotated in the 5′UTRs of the canonical transcripts of twelve iGluR genes. Nevertheless, we found virtually no genetic variations near any of these uATGs in SCH, which are thus unaffected. In the 5′UTR of GRIA3, the alternative allele of the variant rs58044961 was present on all chromosomes in both the SCH subjects and the controls. The variant rs58044961 results in −2G duplication, but the consensus Kozak motif (RccATGG) is not disrupted or attenuated (GgCATGG instead of AgCATGG), so no effects on gene expression are expected.

**Figure 1:**
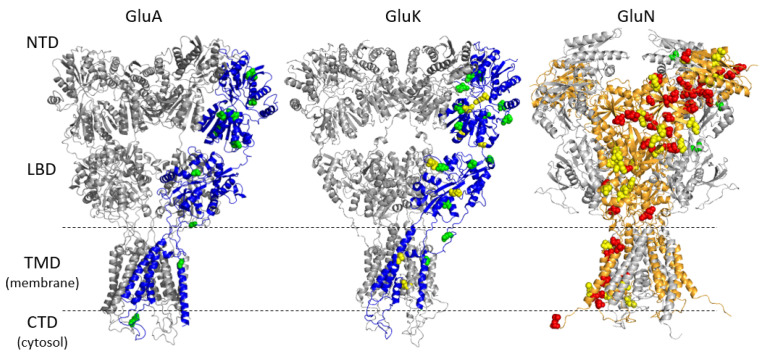
Positions of missense variants found in iGluR subunits. The structures of GluA and GluK represent a prototypical homomeric receptor. Green and yellow residues in the GluA or GluK subunit (in blue) mark missense variants. The structure of GluN represents the heteromeric iGluR (obligatory GLuN1 subunits in gray, variable GluN2/N3 subunits in orange). Missense variants in the GluN1 subunit are depicted in green. Yellow and red residues in the GluN2/N3 subunit (in orange) mark missense variants found in some of the six subunits.

Thirty-four percent of the exonic variants were missense mutations located predominantly in the N-terminal domain (NTD) and intracellular regulatory C-terminal domain (CTD) of glutamate receptors, suggesting a potentially less deleterious effect on the ion channel function (Figure 1). The observed missense variants largely represented benign population variability; even common premature stop gain-of-frameshift variants, which have a negative effect on protein expression of the allele, showed no significant difference in frequency between the SCH subjects and the controls (Table 3).

The GRIN3B frameshift variant p.Gly466AlafsTer18 (rs10666583) was detected in five SCH and three control homozygotes, consistent with the expected genotype frequencies according to the Hardy–Weinberg equilibrium. The second common GRIN3B frameshift variant p.Lys738GlnfsTer34 (rs545736648) is located in a GC-rich region, which complicates its robust variant calling. Based on the 50 bp rule [47], we hypothesize that both rs10666583 and rs545736648 frameshift variants are removed by nonsense-mediated decay (NMD). The influence of the p.Leu11ProfsTer13 (rs3841128) frameshift variant from the first exon of GRIA1, which was detected in seven SCH patients (six heterozygotes and one homozygote) and five control heterozygotes, remains to be elucidated. The GRIN2C frameshift variant p.Lys788ThrfsTer13 (rs754674133) from the twelfth exon was detected in one patient and is scored as “likely pathogenic” according to the ACMG classification. The novel nonsense variant (p.Gln56*) from the first exon of GRIN3A was scored as “pathogenic” according to the ACMG classification and was observed without co-occurrence with a common frameshift variant. The twenty exonic variants listed in Table 3 represent those with predicted deleterious effects. Missense variants were assessed using at least five prediction tools and must have a CADD_phred value above 20. Of the eighteen exonic variants unique to SCH, nine were classified as deleterious. The one novel missense variant we found, p.Pro1386Leu from the last exon of GRIN2A (hg38; chr16:9,763,387), was classified as benign. In addition, of the eight exonic variants present only in the controls, five were also classified as deleterious. 

With few exceptions, the variants significantly associated with SCH were located in intronic regions (Supplementary Figure S1). These variants may represent deep intronic variants with a splicing effect that is difficult to assess or variants that are in linkage disequilibrium with the functional variants. 

### 3.2. Genetic Variation Associated with FES

**Figure 2:**
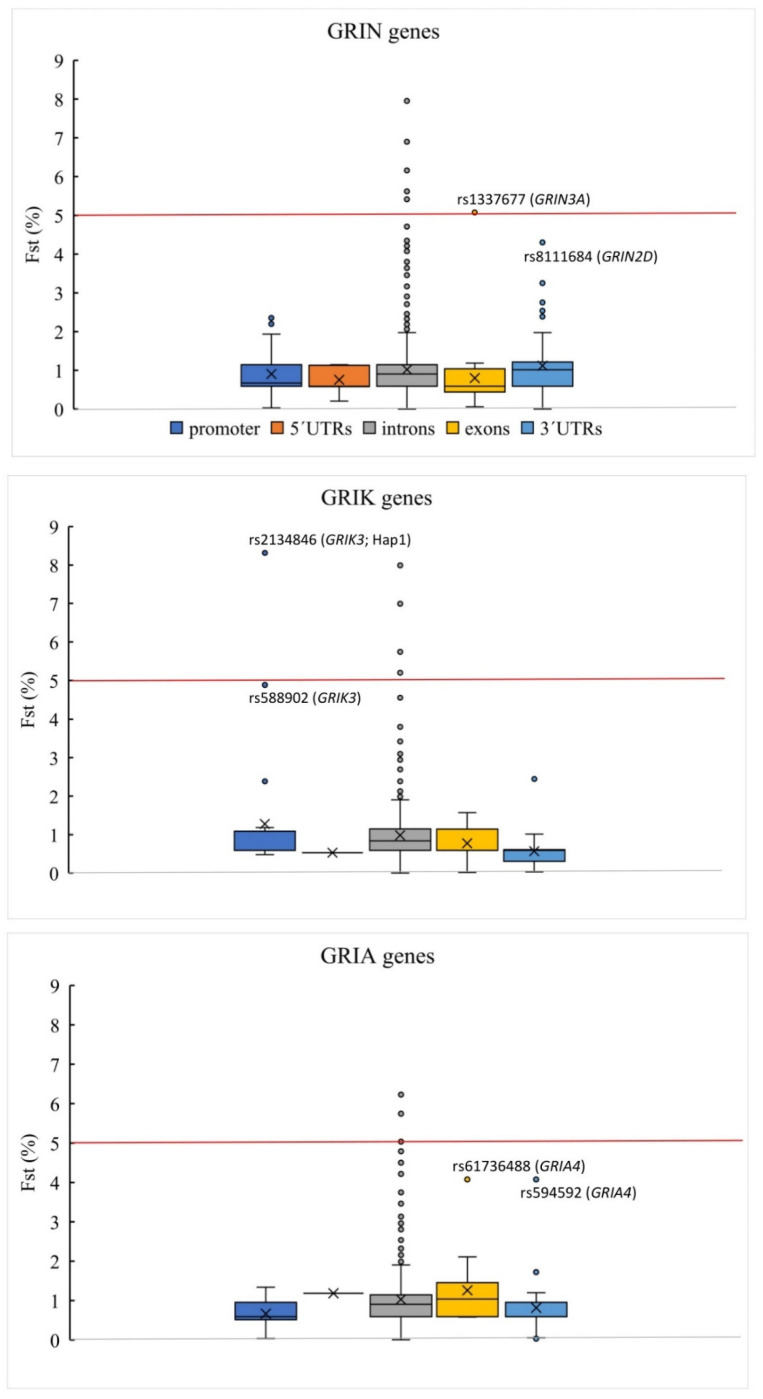
The Fst (%) estimate for variants detected in iGluR genes. Boxplots show variants with Fst above 5% (last percentile of the Fst distribution) that are located nearly exclusively in intronic sequences. Non-intronic variants are highlighted. The rs61736488 is a synonymous substitution p.Lys798Lys from exon 15 of *GRIA4* with a medium CADD_phred score of 10.66. The variant rs594592 is located in the 3′UTR of *GRIA4* with a low CADD_pherd score of 1.841. The *GRIK3* promoter variant rs2134846 is part of the haplotype Hap1. Another *GRIK3* promoter variant rs588902 with a high CADD_phred score of 22.3 is highly conserved and has the GERP score of 2.51. The exonic variant rs1337677 is a synonymous variant p.Pro229Pro from the first exon of *GRIN3A* that is in almost complete linkage with the haplotype Hap4 and has a medium CADD_phred score of 11.62. The *GRIN2D* 3′UTR variant rs8111684 has a CADD_phred score of 16.41.

In the context of polygenic inheritance in SCH, we assumed a cumulative effect of the common susceptible variants. We used the fixation index (Fst) estimate to determine the degree of genetic divergence between the two subpopulations [48,49] that can be run even on small sample sizes. Nearly 20,000 variant loci were included for Fst detection. In case–control studies where both groups are from the same population, Fst detects differences at loci that could be associated with the development of the relevant complex phenotype. The weighted Fst estimate for a subpopulation of SCH cases and controls was −0.001 according to Weir and Cockerham [50]. The negative value indicated a slight excess of heterozygotes (usually caused by the presence of rare variants), but conclusively this analysis of variance did not detect any difference in genetic diversity between the two groups, consistent with their origin from the identical population. For the majority of the variants analyzed (94%), the Fst estimate was less than 2%. Only variants from the last percentile of the estimated variance difference distribution (divergence greater than 5%) became the object of our further interest. These variants were predominantly located in the intronic regions (Figure 2). The higher diversity in the controls was expected as they are derived from a larger population pool than the SCH subjects so only the variants with achieved divergence and higher frequency in SCH were further analyzed.

Loci-specific Fst estimate revealed 189 variants. After excluding 107 variants with higher frequency in the controls than in the SCH subjects, 82 variants remained to be further analyzed and might be associated with schizophrenia. Most of them were distributed among the nine different haplotypes. Certain variants seem to be driven by these haplotypes, and their increased frequency in SCH appears to be due to the observed genetic linkage. Data from the GRIA3 located on the X chromosome were excluded because the population genetics of sex chromosomes differ. However, we found a single haplotype TAAAAAGTT from intron twelve of GRIA3 (hg38; chrX:123,436,446-123,444,102) with a mean Fst = 0.061. The remaining eight haplotypes with detailed characterization are listed in Table 4. All haplotypes (labeled Hap1–8) associated with SCH are located in the intronic sequences. The Hap1 haplotype included regions with strong H3K27Ac histone modification, CpG island, curated transcription factor binding sites (OREG1520167; OREG1520175; OREG1684636), and candidate enhancer and promoter-like signatures. The rs59729868 from Hap1 is located in close proximity to the core-binding sequence for the transcription factor SPI1. The rs2485530 from Hap4 resides in the curated transcription factor binding site (OREG1369890; OREG1345162) within the recognized insulator element in close proximity to the CTCF binding sequence. The rs1939673 from Hap5 is assigned to the curated transcription factor binding site (OREG1938711), which was evaluated as an enhancer element. The rs2284412 from Hap7 is located in a region rich in H3K4me3 modifications (UCSF Brain DNA Methylation) that is co-occupied by multiple candidate transcription factor binding sites. 

Besides the nine haplotypes, four additional SNPs were revealed based on the Fst estimate (Table 4). All of these SNPs were also intronic, had low conservation score, and were not located within regulatory sequences or DNaseI hypersensitivity sites. Therefore, they were not included in the proposed scoring model for estimating cumulative risk.

### 3.3. Scoring Model for the Estimation of Cumulative Risk 

**Figure 3:**
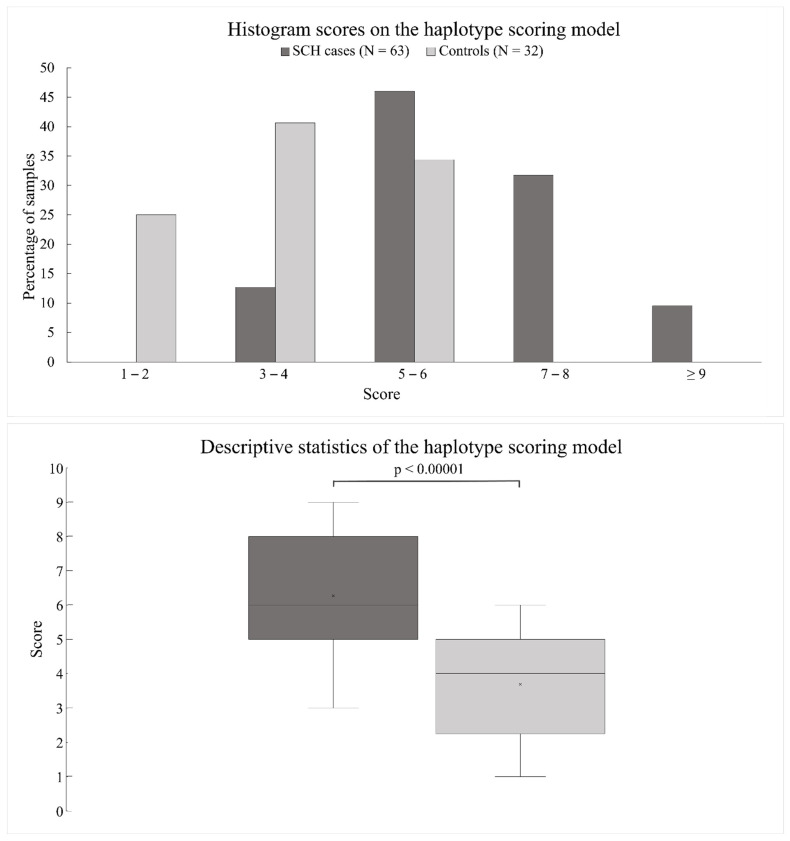
Score histograms and descriptive statistics of the haplotype scoring model. The top graph shows a histogram (%) of the scores distribution in both groups; SCH subjects (dark gray) and controls (light gray). In both groups, the haplotype scoring model did not deviate from the normal distribution. The bottom graph shows a boxplot of the scores in the SCH patients (dark gray) and the controls (light gray). There was a significant difference between the medians of the two groups (*p* < 0.00001). The mean score was 6.27 ± 1.58 in the SCH subjects and 3.69 ± 1.40 in the controls.

The polygenic trait is characterized by numerous variants, each contributing with a relatively small effect. These variants could be common and the predisposition to the phenotype is only an extreme combination of normally distributed genotypes from multiple risk loci. In our study, the value of the likelihood ratio (LR) for a specific haplotype suggests an additive risk model for most haplotypes. Therefore, to estimate a possible cumulative effect of discovered haplotypes on the emergence of SCH, a haplotype scoring model was proposed. The presence of a risk haplotype was penalized by one point for heterozygotes and two points for homozygotes. The Kolmogorov–Smirnov test of normality showed a normal score distribution in both the SCH subjects and the controls (*p*-values 0.0844 and 0.2616, respectively). The descriptive statistics of the scoring model is summarized in Figure 3. The medians of a given score in the SCH subjects and the controls were then compared. One-tailed Mann–Whitney U test detected a significant difference in the median of both groups (*p* < 0.00001). The scoring model was used as a simple test to detect SCH. The score of 6 was set as the cutoff point between the positive and the negative test. A positive test is interpreted as a genetic predisposition to schizophrenia. The sensitivity of the test was 0.67, but specificity was high, 0.93. The positive likelihood ratio for the scoring model test reached 7.11. The lower sensitivity of the test is consistent with the expected diversity of disease pathogenesis. According to our data, 9.4% (3 of 32 in our control group) of individuals from the Czech population would test positive. The portion of SCH with a score below six points was 33%.

### 3.4. Correlation of the BrainAGE Index with the Haplotype Scoring Model

**Figure 4:**
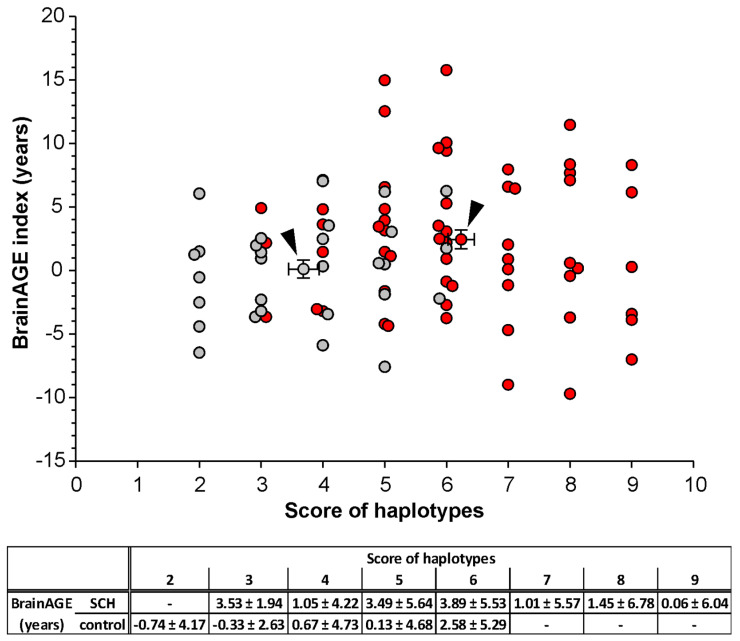
BrainAGE index and haplotype scoring model. The graph shows the distribution of haplotype scores and BrainAGE indexes in both groups; SCH (red) and controls (gray). The arrows mark the mean haplotype score 6.27 ± 1.58 and mean BrainAGE index 2.44 ± 5.54 in SCH, and score 3.69 ± 1.40 and BrainAGE 0.14 ± 3.94 in controls, respectively. The bottom table shows the mean values of BrainAGE index for each scores in both groups.

We supplemented our study with post hoc analyses of gray matter loss using the BrainAGE index, a multivariate pattern recognition technique that quantifies age-related brain tissue loss by employing kernel regression methods in a large training database [51,52]. The SCH patients had an increased mean BrainAGE index of 2.44 ± 5.54 years while the control group had a mean BrainAGE index of 0.14 ± 3.94 years compared to their chronological age. There was a significant difference between the means of the two groups (*p* < 0.05). The mean score of haplotypes in the SCH patients (6.27 ± 1.58) was also higher than in the control group (3.69 ± 1.40), indicating a positive correlation between the overall higher biological brain age and the assumed cumulative effect of the discovered haplotypes in the SCH patients (Figure 4). 

## 4. Discussion

In this study, genes of all the sixteen ionotropic glutamate receptors were sequenced and analyzed in a cohort of Czech SCH patients and matched healthy controls. *GRIN2A*, *GRIN2B*, *GRIN3A*, *GRIN3B*, and *GRIK4* showed increased genetic variability compared to the other iGluR genes. In addition, eight intronic haplotypes were recognized that were significantly associated with SCH. A haplotype scoring model was proposed and used as a simple test to indicate genetic predisposition to schizophrenia. Several missense and frameshift variants were detected. None of these observed and previously annotated variants [53] were associated with SCH.

Recent GWAS studies have identified multiple risk loci associated with SCH [34,35,36]. Published data showed significantly increased genetic variation in genes of the excitatory glutamatergic signaling [37,38,39,40] and confirmed previous pharmacological and genetic studies linking SCH to dysfunction of iGluRs [6]. Previous studies focused on the coding parts of the corresponding genes, where a spectrum of genetic variants was discovered. However, the majority of variants identified were only common polymorphisms, and even those classified as rare missense mutations could only explain isolated SCH cases. The studies mapping the promoters and UTRs of *GRIN2A* and *GRIN2B* described a possible effect of noncoding variants on the emergence of SCH [23,24,32]. Much less is known about intronic variants or cumulative effects of variants.

In our study, overall increased genetic variability was in the SCH patients compared with the control subjects. However, most of these variants were rare (MAF ≤ 0.016 in the SCH cases) and randomly distributed. A significantly different frequency between samples from the SCH subjects and the controls was found for the variants located predominantly in intronic sequences, with a few in 3′UTRs or promoter regions. Our data are consistent with the previously published GWAS studies on 108 genomic loci associated with schizophrenia [34] as well as other studies that have repeatedly shown that intronic variants are significantly associated with SCH.

The identified missense variants that were estimated to be deleterious using the available prediction tools were mostly located in the NTD and CTD of the receptor, suggesting rather minor effects on the receptor function. However, a change in the receptor function, its biogenesis, or localization cannot be completely excluded [9]. Nevertheless, rare missense variants have been detected only in single SCH patients. The estimated pathogenic effect can be exerted on the individual level, but from the population perspective, these variants are not crucial. The polymorphic frameshift variant from exon 3 of *GRIN3B* (rs10666583) had a similar frequency in the SCH subjects and the controls, consistent with the gnomAD allele frequency (MAF_CGTT_ in non-Finnish Europeans is 0.300). The expected frequency of homozygotes in the European population is approximately 9%, well above the lifetime prevalence of 0.5% in SCH [1,2]. The variant rs10666583 was of particular interest as previous studies indicated it as a causal variant and suggested that the truncated protein is produced and interferes with the activity of the wild-type variant, which would have a high impact on the phenotype [54,55]. However, these studies did not address the high frequency of the aberrant allele in the general population. By applying the 50 bp rule, we assumed that the premature stop codon determines the transcript to NMD machinery and acts as a null allele. Recent evidence shows that products of NMD can modulate the transcription of other genes and exert the so-called “genetic compensation response” enabling compensation for allele loss [56]. Thus, the frameshift variant rs10666583 might contribute to the population diversity of NMDAR expression and function but does not play a crucial role in the predisposition to SCH. The same appears to be true for the second common frameshift variant of *GRIN3B* (rs545736648). The effects of a common *GRIA1* frameshift variant rs3841128 and a novel nonsense variant in *GRIN3A* p.Gln56* are rather unclear. The premature stop codon within the first exon can results in either NMD or activation of the new downstream start codon, with all the possible consequences this may have. The variant p.Lys788ThrfsTer13 (rs754674133) from the twelfth exon of *GRIN2C* observed in one SCH patient satisfies the 50 bp rule and should therefore escape the NMD machinery. Therefore, we assume its distinct pathogenicity. The variant was previously described as a rare loss-of-function mutation contributing to SCH [30]. The difference in the frequency of co-occurrence of common frameshift variants in the SCH subjects and the controls did not reach statistical significance (*p*-value = 0.1853), probably due to the small sample size. Nevertheless, the co-occurrence of more than one frameshift variant in two different iGluR genes could represent a risk factor for the development of SCH. 

The 5′UTR sequences did not show remarkable variability, and the extent of genetic variation was similar to that in the exonic regions. This is consistent with the critical role of 5′UTRs in determining the mRNA stability and translational efficiency [57]. The higher rate of variability of 3′UTRs, despite their important role in the regulation of mRNA stability and localization, likely reflects the alternation of sequences under strong negative selection with those under mild selection [58]. Previous studies have shown that genetic variation of 3′UTR of NMDAR genes may have an impact on miRNA binding, which could repress the process of translation [59,60]. All the 3′UTR variants identified in our study were assigned to be potentially nonpathogenic (Figure 2). The C allele of rs890 from the 3′UTR of *GRIN2B*, previously associated with SCH (Chinese Han population), was more frequent in our controls (0.609) than in the SCH subjects (0.460), consistent with gnomAD allelic frequencies in Europeans (0.512). The C allele alters the sequence targeted by miR-4328 and creates a new miRNA site (miR-1468-5p) resulting in the repression of transcript activity [59]. However, it is quite difficult to assess functionality of the variants from 3′UTR sequences, as a number of proteins bind coordinately to 3´UTR elements, often in a tissue-specific manner.

For the given canonical transcripts, most of the observed genetic variation was located within introns. Fst calculation was used to identify those intronic variants associated with SCH. Strict constraints were applied, and only variants from the last percentile of the Fst distribution were further analyzed. The Fst estimate is used to detect population substructure due to inbreeding or genetic drift. To our knowledge, the SCH subjects were unrelated individuals recruited from the area of more than 1 million people with a generally high turnover of persons from all over the Czech Republic. Using Fst calculation, eight intronic haplotypes and four intronic SNPs with significantly higher frequency were detected in the SCH subjects compared to the controls. A highly conserved intronic nucleotide may highlight a splicing element, secondary structure-forming region, or enhancer element or affect protein abundance by some other mechanism [61]. Almost all of these haplotypes contain transcription factor binding sites and curated or putative regulatory elements. Haplotypes Hap1, Hap2, Hap4, and Hap8 are located at the 5′end (promoter, intron 1 or 2) of *GRIK3*, *GRIA1*, *GRIN3A*, and *GRIK1* genes, respectively, and may have a direct impact on the efficiency of transcription initiation. The most interesting haplotype is Hap1 of *GRIK3*, as it combines a region with strong H3K27Ac histone modification, CpG island, and promoter-like signatures. GRIK3 encodes the GluK3 subunit, which plays a role in presynaptic facilitation of signal transduction in hippocampal mossy fibers [62]. In the event that Hap1 negatively affects the *GRIK3* promoter, lower GluK3 expression could contribute to glutamatergic hypofunction in individual cases. Analysis of the *GRIK3* promoter revealed another variant c.-1420G>T (rs588902) that was not in linkage with the Hap1 haplotype but had a high Fst estimate (0.049). Previously, common polymorphic variants from the promoter regions of *GRIA1*, *GRIA3*, *GRIN1*, or *GRIN2A* were associated with susceptibility to addiction, migraine, or schizophrenia [32,63,64,65]. Gene transcription requires the RNA polymerase complex II bound to the core of the promoter, which is decorated by regulatory factors that recognize specific DNA sequences [66]. Polymorphisms within promoter regions can modify binding motifs and alter promoter activity [67]. In the SCH subjects, the length of the microsatellite (GT)_n_ repeat (rs3219790) within the *GRIN2A* promoter differed significantly from the controls and was associated with a change in transcriptional activity [32]. Similarly, the minor A allele of the c.-447-42G>A polymorphism (rs3764030) from the *GRIN2B* promoter was assigned using reporter gene assays as a gain-of-function variant associated with an increase in the *GRIN2B* mRNA levels [68]. The frequency of the A allele in the SCH subjects (0.294) was not significantly different (*p*-value = 0.1805) from that in the controls (0.203) and the gnomAD genomes frequency in the European population (0.190). The Fst estimate for rs3764030 was 0.012 (GERP = −1.74; CADD_phred = 17.16). Our data do not contradict the assigned influence of the A allele on *GRIN2B* expression but indicate its possibly small contribution to the emergence and development of SCH.

Since the likelihood ratios for individual haplotypes follow the simple additive model, we proposed a haplotype scoring model to estimate a possible cumulative effect in SCH. The scoring model was used as a simple test where a score of 6 indicates test positivity interpreted as a genetic predisposition to schizophrenia. With a lifetime prevalence of schizophrenia of 4.8/1000 [2], the population risk is approximately 0.5%. Test positivity could increase the lifetime risk of schizophrenia up to 3.3%. Such a test is not intended for population diagnosis, as only susceptibility to schizophrenia is inherited, but could be helpful in diagnosing the familial form of the disorder or pathogenesis. Diverse polygenic risk scores (PRSs) are beginning to be an effective tool for assessing familial risk of polygenic traits or response to treatment [69,70,71]. The estimated prevalence of score 6 in the Czech population is 9%, with 3.3% probability of schizophrenia in this group, this could explain about 60% of the lifetime prevalence of SCH in the affected Czech population. In 33% of FES patients with a score below six points, the pathogenesis of schizophrenia is not predominantly associated with the variation of iGluR genes or at least with the estimated risk haplotypes. However, such association of the defined haplotypes with SCH and the proposed test validity need to be verified in a larger group of SCH patients and controls. 

A common sign of SCH is exaggerated brain maturation with age-altered loss of gray mater. The BrainAGE aggregates the complex multidimensional aging patterns in a single value, i.e., the deviation of brain atrophy from normal brain aging, expressed in years [72]. BrainAGE can be estimated either for the aggregated whole brain data or for individual tissues. A longitudinal neuroimaging study examining progressive brain tissue loss in schizophrenia patients revealed a brain age gap of 3.36 ± 5.87 years at baseline (the first MRI scans) compared to the chronological age of the patients [73]. In the study that focused on patients with early stages of SCH and bipolar disorder, a higher brain age (2.64 ± 4.15) than their chronological age was estimated [74]. Our group of FES patients had a brain age gap of 2.44 ± 5.54, similarly to previous studies, and this value correlated positively with a higher mean haplotype score in SCH. The largest difference in brain age between the SCH group and the control group was for two haplotype scores 3 and 5. In contrast, for a given haplotype score 4 or 6, we observed smaller differences in the brain age gap between the SCH group and the control group than should correspond to the estimated values of the mean in the SCH subjects and the controls. We suppose that this was caused by a small number of samples (n ≤ 6) within the specific score combined with genetic heterogeneity. This possibility supports studies showing that individual changes in the GM volume are influenced by the previous course of psychosis, medication, and other genetic or environmental factors [75,76,77].

Our data support the concept that genetic predisposition to SCH may also result from the cumulative effect of a number of polymorphisms, each contributing with a small impact. In our study of iGluRs, such polymorphic loci were located in conserved intronic sequences rather than in exonic ones. Variability in the regulatory elements of genes can affect the orchestration of gene activity and result in altered regulation of protein expression. 

The study estimates genetic variability in iGluRs plays a meaningful role in two-thirds of FES patients. Genetic variability associated with SCH was found for variants in intronic sequences and variants in 3′UTRs or promoter regions, suggesting altered mRNA processing and lower gene expression that could possibly be compensated in certain cases by the administration of positive modulators of glutamatergic signaling.

## 5. Conclusions

In this study, we investigated the whole genes, inclusive introns, and extended promoters encoding all sixteen ionotropic glutamate receptors to capture the overall distribution of sequence variants in a cohort of SCH patients and matched controls. The Fst estimate highlighted eight intronic haplotypes significantly associated with FES patients in our study. These haplotypes were used in the proposed scoring model and moreover as a simple test where a score of 6 indicated positivity and was interpreted as a genetic predisposition to SCH. The positive likelihood ratio for the scoring model test reached 7.11. Genetic variability significantly associated with SCH was found for variants predominantly in intronic sequences, with few variants in 3′UTRs or promoter regions and none in 5′UTRs or exonic parts of the genes studied. Our data suggest that some intronic regulatory regions of iGluR genes and their common variability could be one of the components from which the genetic predisposition to SCH is composed.

## Figures and Tables

**Table 1:** Sociodemographic and clinical Data for the first-episode schizophrenia-spectrum and healthy control groups.

	FES (n = 63)	HC (n = 32)	*p*-Value (Two-Tailed *t*-Test)
Age, years; mean (SD)	24.62 (6.94)	27.84 (6.96)	0.0355
Female, No. (%)	24 (38.10)	17.00 (53.13)	0.16
Male, No. (%)	39 (61.90)	15.00 (46.88)	
Education, years; mean (SD)	12.72 (2.84)	16.74 (2.40)	0.00 +
Schizophrenia, No. (%)	38 (60.32)		
Other schizophrenia-spectrum disorders *, No. (%)	25.00 (39,68)		
PANSS Positive Subscale; mean (SD)	14.82 (4.91)		
PANSS Negative Subscale; mean (SD)	18.48 (6.46)		
PANSS General Psychopathology Subscale; mean (SD)	36.17 (8.87)		
PANSS total; mean (SD)	69.48 (17.35)		
Duration of untreated psychosis (months); mean (SD)	3.61 (5.05)		
Age at disease onset; mean (SD)	24.13 (6.97)		
Chlorpromazine equivalents, mg/d; mean (SD)	399.67 (192.03)		
Duration of antipsychotic treatment (months); mean (SD)	1.79 (2.96)		

Note: FES, first-episode schizophrenia-spectrum patients; HC, healthy controls; PANSS, Positive and Negative Symptom Scale; SD, standard deviation; +, Pearson’s chi-squared test; *, the ICD-10 diagnosis of acute and transient psychotic disorders is congruent with the DSM-IV-defined brief psychotic disorder.

**Table 2:** Genetic variations of iGluR genes. The summary of detected genetic variations within the promoter regions (1500 bp upstream of a TSS), 5′UTRs, exons, introns, and 3′UTRs of sixteen iGluR genes. The rate of variation in specific parts of the genes is expressed as the frequency of variants per 1000 bp. The RefSeq transcript corresponds to the canonical Ensembl transcript. Note: the RefSeq transcript does not necessarily have to be a canonical transcript itself in the hg38 assembly database.

Sample	Chr	iGluR Gene	RefSeq	Ensembl Canonical	Promotor (Existing/Novel)	Per 1000 bp	5´UTR (Existing/Novel)	Per 1000 bp	EXONS (Existing/Novel)	Missense/Synonymous (Novel)	Frameshift Variant	Per 1000 bp	INTRONS (Existing/Novel)	Per 1000 bp	3´UTR (Existing/Novel)	Per 1000 bp
SCH cases	chr5	GRIA1	NM_001258022.1	ENST00000518783.1	10/0	6.7	0/0	0.0	3/0	0/2	1	1.1	(1563) 1522/41	4.9	4/1	2.0
Controls	chr5	GRIA1	NM_001258022.1	ENST00000518783.1	8/0	5.3	0/0	0.0	3/0	1/1	1	1.1	(1423) 1396/27	4.5	3/0	1.2
SCH cases	chr4	GRIA2	NM_000826.4	ENST00000296526.7	1/0	0.7	0/0	0.0	3/0	1/2	0	1.1	(349) 333/16	2.5	8/1	3.4
Controls	chr4	GRIA2	NM_000826.4	ENST00000296526.7	2/0	1.3	1/0	3.1	2/0	0/2	0	0.8	(302) 297/5	2.2	6/0	2.3
SCH cases	chrX	GRIA3	NM_000828.4	ENST00000622768.4	4/0	2.7	0/0	0.0	2/0	1/1	0	0.7	(540) 526/14	1.7	6/0	2.7
Controls	chrX	GRIA3	NM_000828.4	ENST00000622768.4	2/1	2.0	0/0	0.0	1/0	0/1	0	0.4	(446) 440/6	1.5	1/0	0.5
SCH cases	chr11	GRIA4	NM_000829.4	ENST00000282499.1	5/1	4.0	1/0	2.3	4/0	0/4	0	1.5	(1107) 1068/39	3.0	9/0	3.8
Controls	chr11	GRIA4	NM_000829.4	ENST00000282499.1	4/0	2.7	1/0	2.3	2/0	0/2	0	0.7	(948) 932/16	2.6	8/0	3.4
SCH cases	chr21	GRIK1	NM_001330994.2	ENST00000327783.4	4/0	2.7	0/0	0.0	4/0	1/3	0	1.4	(1620) 1589/31	4.1	1/0	3.4
Controls	chr21	GRIK1	NM_001330994.2	ENST00000327783.4	1/0	0.7	0/0	0.0	4/0	0/4	0	1.4	(1339) 1317/22	3.4	0/1	3.4
SCH cases	chr6	GRIK2	NM_021956.4	ENST00000421544.1	0/0	0.0	0/0	0.0	4/1	1/4 (1)	0	1.8	(2816) 2756/60	4.2	5/0	3.2
Controls	chr6	GRIK2	NM_021956.4	ENST00000421544.1	0/0	0.0	0/0	0.0	4/0	1/3	0	1.5	(2339) 2307/32	3.5	3/0	1.9
SCH cases	chr1	GRIK3	NM_000831.4	ENST00000373091.8	5/0	3.3	0/0	0.0	3/0	1/2	0	1.1	(938) 912/26	4.1	13/0	2.1
Controls	chr1	GRIK3	NM_000831.4	ENST00000373091.8	6/0	4.0	0/0	0.0	2/0	1/1	0	0.7	(602) 592/10	2.6	4/0	0.6
SCH cases	chr11	GRIK4	NM_014619.4	ENST00000527524.2	10/1	7.3	1/0	3.5	7/0	1/6	0	2.4	(1976) 1938/38	4.2	10/0	3.8
Controls	chr11	GRIK4	NM_014619.4	ENST00000527524.2	6/0	4.0	1/0	3.5	6/0	1/5	0	2.1	(1429) 1397/32	3.0	8/0	3.0
SCH cases	chr19	GRIK5	NM_001301030.1	ENST00000301218.4	3/0	2.0	0/0	0.0	2/0	1/1	0	0.7	(65) 63/2	1.1	1/0	3.0
Controls	chr19	GRIK5	NM_001301030.1	ENST00000301218.4	3/1	2.7	0/0	0.0	2/1	2/1 (1)	0	1.0	(50) 49/1	0.8	1/0	3.0
SCH cases	chr9	GRIN1	NM_001185090.2	ENST00000371553.3	2/0	1.3	0/0	0.0	3/0	0/3	0	1.1	(67) 67/0	2.6	0/0	0.0
Controls	chr9	GRIN1	NM_001185090.2	ENST00000371553.3	2/0	1.3	0/0	0.0	2/0	0/2	0	0.7	(60) 56/4	2.4	0/0	0.0
SCH cases	chr16	GRIN2A	NM_001134407.3	ENST00000330684.3	28/0	18.7	0/0	0.0	7/1	(1) 4/4	0	1.8	(2162) 2112/50	5.2	31/0	3.2
Controls	chr16	GRIN2A	NM_001134407.3	ENST00000330684.3	17/0	11.3	0/0	0.0	6/0	4/2	0	1.4	(1842) 1816/26	4.5	29/0	3.0
SCH cases	chr12	GRIN2B	NM_000834.4	ENST00000609686.1	4/0	2.7	2/2	19.1	12/1	0/13 (1)	0	2.9	(1779) 1730/49	4.3	134/1	6.0
Controls	chr12	GRIN2B	NM_000834.4	ENST00000609686.1	4/0	2.7	2/0	9.5	10/0	0/10	0	2.2	(1449) 1430/19	3.5	107/1	4.8
SCH cases	chr17	GRIN2C	NM_000835.5	ENST00000293190.5	1/0	0.7	1/0	6.8	6/0	3/2	1	1.6	(31) 30/1	2.3	0/0	0.0
Controls	chr17	GRIN2C	NM_000835.5	ENST00000293190.5	1/1	1.3	0/0	0.0	7/0	3/4	0	1.9	(27) 27/0	2.0	1/0	2.4
SCH cases	chr19	GRIN2D	NM_000836.2	ENST00000263269.3	5/0	3.3	1/0	11.4	3/0	0/3	0	0.8	(109) 107/2	2.4	1/0	1.0
Controls	chr19	GRIN2D	NM_000836.2	ENST00000263269.3	2/0	1.3	1/0	11.4	3/0	1/2	0	0.8	(87) 84/3	1.9	1/0	1.0
SCH cases	chr9	GRIN3A	NM_133445.2	ENST00000361820.3	6/0	4.0	6/0	10.0	18/0	8/10	0	5.4	(760) 752/8	4.7	14/1	3.9
Controls	chr9	GRIN3A	NM_133445.2	ENST00000361820.3	4/0	2.7	5/0	8.3	15/0	6/9	0	4.5	(603) 597/6	3.7	13/2	3.9
SCH cases	chr19	GRIN3B	NM_138690.3	ENST00000234389.3	13/2	10.0	0/0	0.0	25/1	16/9 (1)	1	8.3	(38) 36/2	6.3	0/0	0.0
Controls	chr19	GRIN3B	NM_138690.3	ENST00000234389.3	11/0	7.3	0/0	0.0	19/0	10/9	0	6.1	(29) 29/0	4.8	0/0	0.0

**Table 3:** Missense variants of iGluR genes with assigned deleterious effect. The list shows nonsense, frameshift, and missense variants of iGluR genes. Missense variants were classified as deleterious by at least five prediction tools and must have a CADD_phred value above 20. The corresponding minor allele frequencies (MAF) in the SCH subjects and the healthy controls with assessed *p*-values are shown (ND = not determined due to low frequency of the variant in both groups).

ID	hg38	Transcript (Ensembl)	Consequence	iGluR Gene	Exon	Ref. Allele	ALT Allele	SCH Cases, MAF	Controls, MAF	*p*-Value *
rs145573036	chr4:157336626	ENST00000296526.12	p.Glu575Gln	GRIA2	11/16	G	C	0.0079	0.0000	ND
rs3841128	chr5:153492228	ENST00000518783.1	p.Leu11ProfsTer13	GRIA1	1/16	–	C	0.0635	0.0781	0.7643
rs146865938	chr5:153650491	ENST00000518783.1	p.Arg218Cys	GRIA1	4/16	C	T	0.0000	0.0156	0.3368
rs71509734	chr9:101573337	ENST00000361820.6	p.Asn1062Ser	GRIN3A	9/9	T	C	0.0079	0.0000	ND
	chr9:101737814	ENST00000361820.6	p.Gln56 *	GRIN3A	1/9	G	A	0.0079	0.0000	ND
rs34755188	chr9:101670973	ENST00000361820.6	p.Arg480His	GRIN3A	3/9	C	T	0.0079	0.0313	0.2628
rs10989589	chr9:101670953	ENST00000361820.6	p.Gly487Arg	GRIN3A	3/9	C	T	0.4048	0.4844	0.3528
rs41297895	chr11:120819909	ENST00000527524.8	p.Ala167Gly	GRIK4	6/21	C	G	0.0000	0.0156	0.3368
rs137906208	chr11:120861999	ENST00000527524.8	p.Arg262His	GRIK4	9/21	G	A	0.0079	0.0000	ND
rs765016248	chr17:74843525	ENST00000293190.10	p.Gln871Arg	GRIN2C	13/13	T	C	0.0159	0.0000	0.5509
rs754674133	chr17:74844496–74844498	ENST00000293190.10	p.Lys788ThrfsTer13	GRIN2C	12/13	TC	–	0.0079	0.0000	ND
rs375174698	chr17:74850716	ENST00000293190.10	p.Arg389Cys	GRIN2C	5/13	G	A	0.0000	0.0156	0.3368
rs545736648 ^#^	chr19:1007862	ENST00000234389.3	p.Lys738GlnfsTer34	GRIN3B	5/9	–	C	0.0246	0.0161	0.7099
rs201638380	chr19:1004804	ENST00000234389.3	p.Glu435Lys	GRIN3B	3/9	G	A	0.0079	0.0000	ND
rs10666583	chr19:1004897	ENST00000234389.3	p.Gly466AlafsTer18	GRIN3B	3/9	–	CGTT	0.3016	0.3125	0.8694
rs566603277	chr19:1005200	ENST00000234389.3	p.Gly567Ser	GRIN3B	3/9	G	A	0.0079	0.0000	ND
rs765625485	chr19:1005375	ENST00000234389.3	p.Ala625Val	GRIN3B	3/9	C	T	0.0000	0.0156	0.3368
rs2285906	chr19:1008684	ENST00000234389.3	p.Ala845Thr	GRIN3B	7/9	G	A	0.2063	0.0938	0.0643
rs61744375	chr19:1008705	ENST00000234389.3	p.Glu852Lys	GRIN3B	7/9	G	A	0.0079	0.0000	ND
rs137867437	chr19:42062790	ENST00000301218.8	p.Ser104Pro	GRIK5	3/19	A	G	0.0000	0.0156	0.3368

^#^ The frameshift variant is in the GC-rich region with low coverage and lower confidence of variant calling; the ALT allele frequency in the Czech popuation is estimated to be 0.02 (non-published data). * The *p*-value was calculated using Fisher’s exact test.

**Table 4:** Haplotypes and SNPs revealed using the Fst estimate. Each haplotype is characterized by its SNPs, location, frequency of risk allele in the SCH subjects and the controls, and the corresponding Fisher exact test’s *p*-value. When possible, the odds risk ratio (OR) was calculated for each haplotype, as well as the likelihood ratio (LR) for specific genotypes. The LR suggests an additive model of the risk allele effect for most haplotypes. Variants that exhibit high conservation or colocalize with some regulatory elements or transcription factor binding sites are shown with appropriate symbols and are considered as functional candidate variants. An additional four SNPs are listed and characterized based on their Fst estimate above 0.05.

iGluR Gene	Haplotype	hg38	rs	Location	Allele	Fst	SCH Cases freq	Controls freq	*p*-Value ^1^	OR	Genotype	LR	Genotype	LR	Genotype	LR
GRIK3	Hap1	chr1:37014543	rs3753776 ^&,§^	intron 1	T	0.071281	0.381	0.188	0.0080	2.67	wt/wt	0.554	wt/Hap1	1.904	Hap1/Hap1	2.270
		chr1:37017453	rs2359647	intron 1	T	0.071281										
		chr1:37023779	rs6686296 ^#^	intron 1	G	0.071281										
		chr1:37028109	rs59729868 ^£^	intron 1	A	0.071281										
		chr1:37031920	rs3845491	intron 1	T	0.083134										
		chr1:37035122	rs2134846	promoter	A	0.083134										
GRIA1	Hap2	chr5:153527505	rs566577 ^¥,£^	intron 2	C	0.062782	0.183	0.047	0.0126	4.54	wt/wt	0.754	wt/Hap2	2.872	Hap2/Hap2	ND
		chr5:153532297	rs1493395	intron 2	G	0.062782										
		chr5:153534826	rs1908100	intron 2	T	0.062782										
GRIK2		chr6:101460819	rs2518230	intron 4	T	0.055046	0.548	0.344	0.0091	2.31	wt/wt	0.473	wt/Hap3	1.227	Hap3/Hap3	2.032
	Hap3	chr6:101462357	rs2245037 ^&^	intron 4	G	0.055046										
		chr6:101467933	rs2579937	intron 4	A	0.056160										
GRIN3A	Hap4	chr9:101711604	rs2485530 ^#,§,¥,£^	intron 2	C	0.050761	0.079	0.000	0.0174	ND	wt/wt	0.841	wt/Hap4	ND	Hap4/Hap4	ND
		chr9:101714448	rs2506363	intron 1	C	0.050761										
		chr9:101715378	rs2210991	intron 1	C	0.055466										
		chr9:101716352	rs2506364	intron 1	A	0.050761										
		chr9:101717714	rs2065965	intron 1	G	0.050761										
		chr9:101720595	rs7849059	intron 1	A	0.050761										
GRIK4	Hap5	chr11:120746795	rs1945010 ^¥,£^	intron 3	C	0.058187	0.683	0.500	0.0177	2.15	wt/wt	0.361	wt/Hap5	0.845	Hap5/Hap5	2.027
		chr11:120747370	rs1939673 ^#,§,¥,£^	intron 3	T	0.051846										
GRIK4	Hap6	chr11:120927187	rs5795249	intron13	T	0.101537	0.222	0.047	0.0016	5.81	wt/wt	0.683	wt/Hap6	3.372	Hap6/Hap6	ND
		chr11:120927920	rs1893816	intron13	T	0.101537										
		chr11:120930476	rs2186620 ^¥,£^	intron13	C	0.094634										
GRIN2B	none	chr12:13634912	rs2160519	intron 4	C	0.057178	0.087 (C)	0.000 (C)	0.0171	ND	T/T	0.825	T/C	ND	C/C	ND
GRIN2B	none	chr12:13722646	rs2110984	intron 3	T	0.080249	0.825 (T)	0.641 (T)	0.0066	2.65	C/C	0.508	C/T	0.481	T/T	1.985
GRIN2B		chr12:13728086	rs2284412 ^#,¥,£^	intron 3	T	0.054135	0.317	0.156	0.0228	2.51	wt/wt	0.669	wt/Hap7	1.419	Hap7/Hap7	ND
	Hap7	chr12:13741902	rs10772710	intron 3	A	0.054135										
		chr12:13744486	rs2268121 ^$,¥,£^	intron 3	C	0.054135										
GRIN2B	none	chr12:13871835	rs10845851	intron 1	G	0.061579	0.095 (G)	0.000 (G)	0.0072	ND	A/A	0.825	A/G	ND	G/G	ND
GRIN2D	none	chr19:48437650	rs56125279	intron 10	G	0.051118	0.190 (G)	0.063 (G)	0.0181	3.53	C/C	0.762	C/G	2.288	G/G	ND
GRIK1	Hap8	chr21:29925689	rs2832486	intron 1	T	0.052022	0.730	0.563	0.0227	2.10	wt/wt	0.308	wt/Hap8	0.789	Hap8/Hap8	1.808
		chr21:29926886	rs2832488	intron 1	T	0.052022										
		chr21:29927152	rs2832489	intron 1	A	0.052022										
		chr21:29929318	rs2832490	intron 1	G	0.052022										
		chr21:29930154	rs2255821	intron 1	A	0.052022										
		chr21:29930365	rs767253 ^¥,£^	intron 1	C	0.052022										
		chr21:29931426	rs2300333	intron 1	G	0.052022										
		chr21:29931851	rs2832492	intron 1	C	0.052022										
		chr21:29933351	rs9982938 ^¥,£^	intron 1	T	0.052022										
		chr21:29933357	rs9982699 ^¥,£^	intron 1	C	0.052022										
		chr21:29933999	rs3831792	intron 1	AATATAAATG	0.052022										
		chr21:29934461	rs2243533	intron 1	T	0.052022										
		chr21:29934499	rs2243535	intron 1	T	0.052022										
		chr21:29934740	rs2243435	intron 1	T	0.052022										
		chr21:29934751	rs2243436	intron 1	C	0.052022										
		chr21:29936109	rs2832494	intron 1	C	0.052022										
		chr21:29937434	rs2832495	intron 1	C	0.052022										
		chr21:29937912	rs2409355	intron 1	C	0.052022										
		chr21:29938441	rs2832496	intron 1	T	0.052022										
		chr21:29938848-29938850	rs66799775	intron 1	G	0.052022										
		chr21:29938853	rs75584618 ^&^	intron 1	T	0.052022										
		chr21:29938854	rs77191337	intron 1	T	0.052022										

^1^ Fisher exact test statistic value. ^&^ highly conservative (GERP > 1.8); ^#^ DNaseI hypersensitivity peak cluster ENCODE (UCSC); ^$^ enhancer (Ensembl); ^§^ regulatory region ORegAnno (UCSC); ^¥^ regulatory region (Ensembl); ^£^ transcription factor binding site (Ensembl); OR = Odds ratio; LR = Likelihood ratio.

## Data Availability

The data supporting the reported results can be found at https://zenodo.org/record/5347495 (accessed on 31 August 2021).

## Supplementary Materials

The following are available online at https://www.mdpi.com/article/10.3390/jpm11121250/s1, Figure S1: The position of the detected variants in the iGluR genes.
