# Supplementary material for: Evidence for the Association between the Intronic Haplotypes of Ionotropic Glutamate Receptors and First-Episode Schizophrenia
Source: J Pers Med. 2021 Nov 25;11(12):1250. doi: 10.3390/jpm11121250 (PMC8708351; doi:10.3390/jpm11121250)
Supplement: Supplementary file 1 [file jpm-11-01250-s001.zip › jpm-1384057-supplementary.pdf]

Supplementary Materials

Supplementary Figure S1. The position of the detected variants in the iGluR genes.

Manhattan plots show the position of each variant in each iGluR gene. Black dots represent variants located in introns and 3′UTRs of genes, while the gray dots indicate variants found in 5′UTRs and exons. Variants above the black horizontal dashed line are potentially associated with SCH based on the significant difference in minor allele frequency between SCH cases and controls (p-value < 0.01).

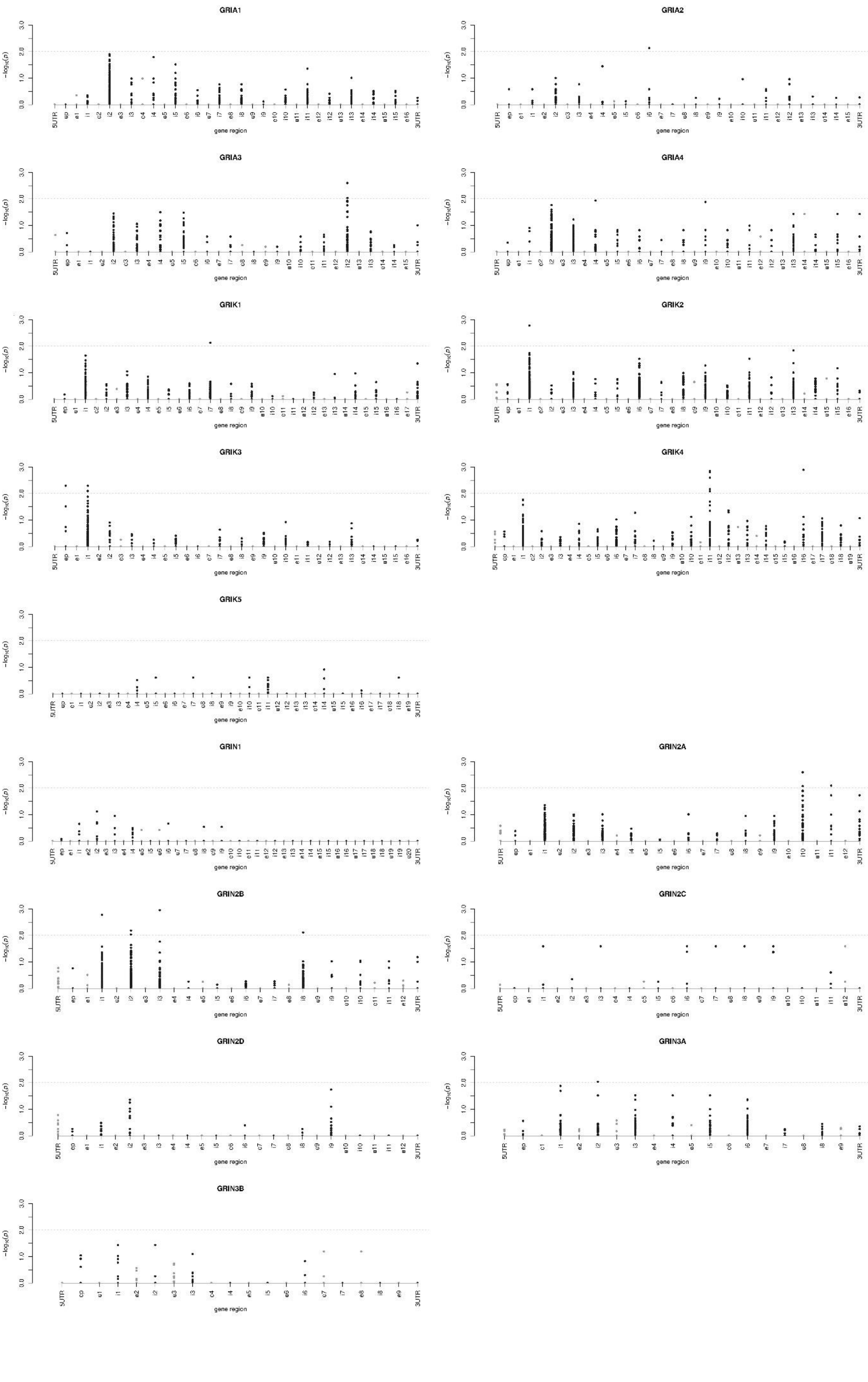
GRIA1
GRIA2
GRIA3
GRIA4
GRIK1
GRIK2
GRIK3
GRIK4
GRIK5
GRIN1
GRIN2A
GRIN2B
GRIN2C
GRIN2D
GRIN3A
GRIN3B
gene region
−log10(p)
